# Predicting the risk of postoperative avascular necrosis in patients with talar fractures based on an interpretable machine learning model

**DOI:** 10.3389/fbioe.2025.1644261

**Published:** 2025-07-31

**Authors:** Jian Zhang, Jihai Xu, Jiapei Yu, Hong Chen, Xin Hong, Songou Zhang, Xin Wang, Chengchun Shen

**Affiliations:** ^1^ Department of Orthopaedics, Ningbo No.6 Hospital, Ningbo, China; ^2^ Ningbo Clinical Research Center for Orthopedics, Sports Medicine and Rehabilitation, Ningbo, China; ^3^ Department of Orthopedics, Zhongda Hospital of Southeast University, Nanjing, China; ^4^ Department of Plastic Reconstructive Surgery and Hand Microsurgery, Ningbo No.6 Hospital, Ningbo, China; ^5^ Department of Foot and Ankle Surgery, Ningbo No.6 Hospital, Ningbo, China; ^6^ Department of Clinical Medicine, Health Science Center, Ningbo University, Ningbo, China

**Keywords:** machine learning, risk factors, prediction model, avascular necrosis, talar fractures

## Abstract

**Purpose:**

This study aims to develop and validate an interpretable machine learning model for predicting avascular necrosis (AVN) following talar fracture, thereby aiding in personalized prevention and treatment.

**Methods:**

A retrospective cohort study included patients undergoing surgical intervention for talar fractures at Ningbo No.6 Hospital between January 2018 and December 2023. Multidimensional data encompassing demographic characteristics, fracture-related variables, surgery-related parameters, and follow-up information were collected. Patients were randomly allocated to the training and testing sets in a 7:3 ratio. Potential risk factors for postoperative AVN were screened using univariate and multivariate logistic regression analyses. Six machine learning algorithms were employed to construct the prediction models. The performance of the prediction model was evaluated utilizing metrics including area under the receiver operating characteristic curve (AUC), calibration curves, decision curve analysis (DCA), accuracy, sensitivity, specificity, positive predictive value (PPV), negative predictive value (NPV), precision, recall, and F1 score. The SHapley Additive exPlanations (SHAP) provided global and local explanations for the optimal model.

**Results:**

A total of 207 patients with talar fractures were enrolled in our study, with 45 (21.74%) developed AVN, and 162 (78.26%) did not. Univariate and multivariable logistic regression identified six independent risk factors including body mass index (BMI), fracture classification, concomitant ipsilateral foot and ankle fractures, smoking, quality of fracture reduction, and fracture type. Performance evaluation demonstrated that Extreme Gradient Boosting (XGBoost model) achieved high AUC values with superior specificity and sensitivity in both the training and testing sets. The SHAP was performed to analyze the relative importance of features within the model visually and illustrate the impact of each feature on individual patient outcomes.

**Conclusion:**

This study successfully developed and validated an interpretable machine learning model incorporating key clinical and surgical variables to predict AVN following talar fractures. The prediction model identified high-risk patients and critical modifiable factors, facilitating personalized prevention strategies to mitigate this severe complication.

## 1 Introduction

Talar fractures are relatively rare injuries, accounting for 0.1%–2.5% of all fractures and 3%–5% of foot and ankle fractures (Saravi et al., 2021). Despite advancements in the diagnosis and treatment of talar fractures, complication rates remain high, and functional outcomes are generally unsatisfactory (Choi et al., 2023). The unique anatomical structure of the talus, characterized by retrograde blood supply via the tarsal canal artery, minimal ligament and tendon attachments, and limited non-articular surfaces resulting in poor vascularization, predisposes it to vascular compromise following high-energy trauma (Kubisa et al., 2024). Consequently, avascular necrosis (AVN) is one of the major complications in patients with talar fractures, with an incidence rate as high as 31.2% (Dodd and Lefaivre, 2015). Patients with early-stage AVN are usually asymptomatic. Consequently, the majority of patients present to the clinic at a late stage with long-term functional impairment that significantly disrupts their quality of life, and ultimately necessitates interventions such as ankle arthrodesis or joint replacement. Therefore, early prediction and identification of risk factors for AVN following talar fractures are critical to optimizing treatment strategies and improving patient outcomes.

Previous studies have reported traditional risk factors for AVN following talar fractures such as high body mass index (BMI) increasing local mechanical stress on the talus and tobacco smoking which impairs local blood supply (Alley et al., 2024). However, most studies are not comprehensive in terms of risk factors, and simple risk factor analysis has limited clinical application. In addition, radiographic examinations such as computed tomography (CT) and magnetic resonance imaging (MRI), are employed to assess vascular integrity to predict AVN (Chen et al., 2014; Kubisa et al., 2024). However, these parameters inadequately reflect the multifaceted and complex pathophysiological processes that contribute to the development and progression of AVN. Consequently, constructing risk models based on comprehensive clinical characteristics to predict AVN following talar fractures can assist clinicians in developing patient-specific management measures and represents a key strategy for AVN prevention.

Machine learning is a subset of artificial intelligence that focuses on the application of algorithms to analyze complex datasets and learn from previous experience, surpassing traditional methods in predicting clinical outcomes (Churpek et al., 2020; Haug and Drazen, 2023). Recent studies have demonstrated the widespread application of machine learning in the field of orthopaedics, such as in the early detection of implant failure and bone nonunion (Harris et al., 2018; Karnuta et al., 2021). However, its application in predicting complications following talar fractures remains underexplored. In addition, machine learning techniques are often considered “black-box” because explaining the decision-making process of the algorithm is complex and challenging (Fanizzi et al., 2024; Hu et al., 2024). The SHapley Additive exPlanations (SHAP), a component of Explainable Artificial Intelligence (XAI), provides transparent explanations of machine learning decisions and elucidates the rationale behind predictions (Wang et al., 2023), thereby addressing the “black-box” limitation by revealing the mechanisms underlying model decisions.

Therefore, this study aimed to develop and validate an explainable prediction model for AVN following talar fracture surgery by leveraging six advanced machine learning algorithms and integrating multidimensional data including patient clinical, radiographic, and operative variables. Subsequently, we evaluated model performance to identify the optimal algorithm and incorporated SHAP analysis to improve interpretability. Our study aimed to provide guidance for surgeons in implementing personalized prevention and treatment strategies by identifying high-risk patients with AVN, and ultimately reduce the morbidity associated with this devastating complication.

## 2 Methods and materials

### 2.1 Study population

This study enrolled patients with talar fractures who underwent surgical interventions at Ningbo No.6 Hospital between January 2018 and December 2023. Inclusion criteria: (1) patients diagnosed with fresh talar fractures (time from injury to surgery <3 weeks); (2) patients who underwent internal fixation; (3) age ≥18 years; (4) patients with complete clinical data and follow-up > 12 months. Exclusion criteria: (1) primary arthrodesis or amputation; (2) previous ankle or foot surgery; (3) severe foot neuropathy or vascular insufficiency; (4) patients with serious clinical or laboratory data missing; (5) incomplete follow-up information. The study was conducted in accordance with the Declaration of Helsinki and approved by the Institutional Review Board of Ningbo No.6 Hospital. The need for individual patient consent was waived by the Institutional Review Board due to the retrospective nature of the study and the use of anonymized data.

### 2.2 Data collection and processing

Baseline variables were selected based on clinical expertise and relevant literature. Clinical data were extracted from electronic medical records and categorized as follows: (1) demographic characteristics including gender, age, ASA class (American Society of Anesthesiologist physical status classification), BMI, hypertension, diabetes, heart disease, smoking, and drinking; (2) fracture-related variables including injury mechanism, fracture side, fracture classification (Hawkins classification for talar neck fractures and Sneppen classification for talar body fractures), fracture type, and concomitant ipsilateral foot and ankle fractures (Srinath et al., 2024). (3) surgery-related parameters including time to surgery, surgical strategy, fixation method, surgical approach, lateral malleolus osteotomy, medial malleolus osteotomy, intraoperative blood loss, operating duration, and quality of fracture reduction. (4) follow-up information including follow-up time and fixation removal. AVN was diagnosed based on radiographic criteria, including sclerosis, cystic changes, or talar collapse observed on postoperative imaging including (plain radiographs, CT, or MRI) (Alley et al., 2024).

### 2.3 Factor screening

The dataset was randomly partitioned into training (70%) and testing (30%) sets. The training dataset was utilized to develop the prediction model, while the test dataset was reserved for independent validation. The variables in the training set were initially screened by univariate logistic regression analyses. Subsequently, the variables meeting the significance threshold (P < 0.05) were included in multivariate logistic regression analyses. Ultimately, the variables that demonstrated statistical significance in the multivariate logistic regression were incorporated into machine learning algorithms for prediction model construction (Du et al., 2025).

### 2.4 Model development and comparison

Six machine learning algorithms were employed in this study including Random Forest (RF), NaiveBayes (NB), Gradient Boosting Machine (GBM), K-Nearest Neighbors (KNN), Extra Trees (ET), and Extreme Gradient Boosting (XGBoost). Hyperparameters were optimized using grid search combined with manual fine-tuning (Supplementary Table S1). The training set was exploited to construct prediction models and the performance of different algorithms was compared (Li et al., 2025).

Receiver Operating Characteristic (ROC) curves were utilized to evaluate the accuracy of each model, with the Area Under the Curve (AUC) serving as a performance metric. Additionally, Decision Curve Analysis (DCA) and calibration curves were plotted to assess the clinical applicability and calibration of the models. Additional performance metrics were evaluated, including accuracy, sensitivity, specificity, positive predictive value (PPV), negative predictive value (NPV), precision, recall, and F1 score (Quan et al., 2024).

### 2.5 Interpretation tools for the model

To address the “black-box” nature of machine learning models, the SHAP (v1.8.5) was implemented using KernelExplainer for model-agnostic interpretation. This approach ranks the importance of input features and provides explanations for model predictions. The SHAP offers both global and local explanations: global explanations provide consistent and accurate attribution values for each feature, indicating their contribution to the final prediction, while local explanations provide a tailored risk assessment for each patient by assessing the contribution of features to an individual prediction.

### 2.6 Statistical analysis

Statistical analysis was performed using Python version 3.11.4, and a significant difference was set as P < 0.05. Continuous variables were analyzed using Student’s t-test or Mann-Whitney U test, while categorical variables were assessed using the chi-square test or Fisher’s exact test, depending on the data distribution.

## 3 Results

### 3.1 Patient characteristics

A total of 207 patients undergoing surgical intervention for talar fractures were enrolled, while 165 patients were excluded based on the inclusion and exclusion criteria (Supplementary Figure S1). Complete case analysis was performed and no imputation or data augmentation was applied. The baseline characteristics of the included patients are summarized in Table 1. Among these patients, 45 (21.74%) developed AVN following talar fractures, and 162 (78.26%) did not.Patients were randomly allocated to a training set (n = 144, 70%) and a test set (n = 63, 30%). Baseline characteristics were comparable between the training and test sets, with no statistically significant differences (Table 2).

**TABLE 1:** Comparison of baseline characteristics between training and testing sets.

Variable	Total (N = 207)	Training set (N = 144)	Testing set (N = 63)	*P* value
Age (years)	43.92 ± 13.81	42.99 ± 13.40	46.05 ± 14.59	0.136
Gender [N(%)]				0.519
Male	165 (79.71)	117 (81.25)	48 (76.19)	
Female	42 (20.29)	27 (18.75)	15 (23.81)	
ASA class[N(%)]				0.380
1	34 (16.43)	21 (14.58)	13 (20.63)	
2	173 (83.57)	123 (85.42)	50 (79.37)	
BMI (kg*m^-2^)	23.89 ± 3.47	23.95 ± 3.42	23.76 ± 3.62	0.715
Hypertension [N(%)]				0.252
No	169 (81.64)	121 (84.03)	48 (76.19)	
Yes	38 (18.36)	23 (15.97)	15 (23.81)	
Diabetes [N(%)]				0.757
No	200 (96.62)	140 (97.22)	60 (95.24)	
Yes	7 (3.38)	4 (2.78)	3 (4.76)	
Heart disease [N(%)]				0.702
No	197 (95.17)	136 (94.44)	61 (96.83)	
Yes	10 (4.83)	8 (5.56)	2 (3.17)	
Smoke [N(%)]				0.509
No	143 (69.08)	102 (70.83)	41 (65.08)	
Yes	64 (30.92)	42 (29.17)	22 (34.92)	
Alcohol [N(%)]				0.951
No	173 (83.57)	121 (84.03)	52 (82.54)	
Yes	34 (16.43)	23 (15.97)	11 (17.46)	
Injury mechanism [N(%)]				0.695
High energy	88 (42.51)	63 (43.75)	25 (39.68)	
Low energy	119 (57.49)	81 (56.25)	38 (60.32)	
Fracture side [N(%)]				0.314
Left	98 (47.34)	72 (50.00)	26 (41.27)	
Right	109 (52.66)	72 (50.00)	37 (58.73)	
Fracture classification [N(%)]				0.163
Hawkins I	5 (2.42)	1 (1.59)	4 (2.78)	
Hawkins II	25 (12.08)	21 (14.58)	4 (6.35)	
Hawkins III	20 (9.66)	9 (6.25)	11 (17.46)	
Hawkins IV	9 (4.35)	7 (4.86)	2 (3.17)	
Sneppen II	46 (22.22)	29 (20.14)	17 (26.98)	
Sneppen III	6 (2.90)	3 (2.08)	3 (4.76)	
Sneppen IV	34 (16.43)	26 (18.06)	8 (12.70)	
Sneppen V	47 (22.71)	34 (23.61)	13 (20.63)	
Talar neck and body fractures	15 (7.25)	11 (7.64)	4 (6.35)	
Fracture type [N(%)]				0.377
Close fracture	185 (89.37)	131 (90.97)	54 (85.71)	
Open fracture	22 (10.63)	13 (9.03)	9 (14.29)	
Concomitant ipsilateral foot and ankle fractures [N(%)]				0.223
No	120 (57.97)	79 (54.86)	41 (65.08)	
Yes	87 (42.03)	65 (45.14)	22 (34.92)	
Time to surgery	7.39 ± 4.32	7.47 ± 4.19	7.21 ± 4.65	0.397
Surgical strategy [N(%)]				0.090
One-stage closed fixation	4 (1.93)	1 (0.69)	3 (4.76)	
One-stage open fixation	194 (93.72)	138 (95.83)	56 (88.89)	
Multi-stage fixation	9 (4.35)	5 (3.47)	4 (6.35)	
Fixation method [N(%)]				0.914
K wire	19 (9.18)	12 (8.33)	7 (11.11)	
Cannulated screw	128 (61.84)	89 (61.81)	39 (61.90)	
Absorbent rod	18 (8.70)	14 (9.72)	4 (6.35)	
Plate	7 (3.38)	5 (3.47)	2 (3.17)	
Plate and Cannulated screw	35 (16.91)	24 (16.67)	11 (17.46)	
Surgical approach [N(%)]				0.918
Single approach	132 (63.77)	91 (63.19)	41 (65.08)	
Combined approach	75 (36.23)	53 (36.81)	22 (34.92)	
Lateral malleolus osteotomy [N(%)]				0.769
No	201 (97.10)	139 (96.53)	62 (98.41)	
Yes	6 (2.90)	5 (3.47)	1 (1.59)	
Medial malleolus osteotomy [N(%)]				0.833
No	187 (90.34)	131 (90.97)	56 (88.89)	
Yes	20 (9.66)	13 (9.03)	7 (11.11)	
Intraoperative blood loss (mL)	52.56 ± 47.16	53.54 ± 50.07	50.32 ± 40.00	0.930
Operating duration (min)	90.22 ± 36.02	91.25 ± 37.86	87.86 ± 31.56	0.706
Postoperative reduction [N(%)]				0.223
Nearly anatomical	180 (86.96)	122 (84.72)	58 (92.06)	
Poor	27 (13.04)	22 (15.28)	5 (7.94)	
Follow up (month)	16.53 ± 5.32	16.55 ± 5.25	16.49 ± 5.53	0.873
Fixation removal [N(%)]				1.000
No	182 (87.92)	127 (88.19)	55 (87.30)	
Yes	25 (12.08)	17 (11.81)	8 (12.70)	

**TABLE 2:** Comparison of baseline characteristics between the Non-AVN and AVN groups.

Variable	Total (N = 207)	Non-AVN (N = 162)	AVN (N = 45)	*P* Value
Age (years)	43.92 ± 13.81	43.76 ± 14.23	44.51 ± 12.29	0.816
Gender [N(%)]				0.128
Male	165 (79.71)	125 (77.16)	40 (88.89)	
Female	42 (20.29)	37 (22.84)	5 (11.11)	
ASA class[N(%)]				0.961
1	34 (16.43)	26 (16.05)	8 (17.78)	
2	173 (83.57)	136 (83.95)	37 (82.22)	
BMI (kg*m^-2^)	23.89 ± 3.47	23.24 ± 3.35	26.23 ± 2.90	<0.001^*^
Hypertension [N(%)]				0.590
No	169 (81.64)	134 (82.72)	35 (77.78)	
Yes	38 (18.36)	28 (17.28)	10 (22.22)	
Diabetes [N(%)]				1.000
No	200 (96.62)	157 (96.91)	43 (95.56)	
Yes	7 (3.38)	5 (3.09)	2 (4.44)	
Heart disease [N(%)]				0.798
No	197 (95.17)	155 (95.68)	42 (93.33)	
Yes	10 (4.83)	7 (4.32)	3 (6.67)	
Smoke [N(%)]				<0.001^*^
No	143 (69.08)	129 (79.63)	14 (31.11)	
Yes	64 (30.92)	33 (20.37)	31 (68.89)	
Alcohol [N(%)]				0.338
No	173 (83.57)	138 (85.19)	35 (77.78)	
Yes	34 (16.43)	24 (14.81)	10 (22.22)	
Injury mechanism [N(%)]				0.578
High energy	88 (42.51)	71 (43.83)	17 (37.78)	
Low energy	119 (57.49)	91 (56.17)	28 (62.22)	
Fracture side [N(%)]				0.687
Left	98 (47.34)	75 (46.30)	23 (51.11)	
Right	109 (52.66)	87 (53.70)	22 (48.89)	
Fracture classification [N(%)]				<0.001^*^
Hawkins I	5 (2.42)	5 (3.09)	0 (0.00)	
Hawkins II	25 (12.08)	21 (12.96)	4 (8.89)	
Hawkins III	20 (9.66)	7 (4.32)	13 (28.89)	
Hawkins IV	9 (4.35)	0 (0.00)	9 (20.00)	
Sneppen II	46 (22.22)	46 (28.40)	0 (0.00)	
Sneppen III	6 (2.90)	6 (3.70)	0 (0.00)	
Sneppen IV	34 (16.43)	34 (20.99)	0 (0.00)	
Sneppen V	47 (22.71)	34 (20.99)	13 (28.89)	
Talar neck and body fractures	15 (7.25)	9 (5.56)	6 (13.33)	
Fracture type [N(%)]				<0.001^*^
Close fracture	185 (89.37)	157 (96.91)	28 (62.22)	
Open fracture	22 (10.63)	5 (3.09)	17 (37.78)	
Concomitant ipsilateral foot and ankle fractures [N(%)]				<0.001^*^
No	120 (57.97)	110 (67.90)	10 (22.22)	
Yes	87 (42.03)	52 (32.10)	35 (77.78)	
Time to surgery	7.39 ± 4.32	7.30 ± 4.08	7.73 ± 5.15	0.908
Surgical strategy [N(%)]				0.002^*^
One-stage closed fixation	4 (1.93)	4 (2.47)	0 (0.00)	
One-stage open fixation	194 (93.72)	155 (95.68)	39 (86.67)	
Multi-stage fixation	9 (4.35)	3 (1.85)	6 (13.33)	
Fixation method [N(%)]				0.063
K wire	19 (9.18)	13 (8.02)	6 (13.33)	
Cannulated screw	128 (61.84)	106 (65.43)	22 (48.89)	
Absorbent rod	18 (8.70)	16 (9.88)	2 (4.44)	
Plate	7 (3.38)	5 (3.09)	2 (4.44)	
Plate and Cannulated screw	35 (16.91)	22 (13.58)	13 (28.89)	
Surgical approach [N(%)]				0.001^*^
Single approach	132 (63.77)	113 (69.75)	19 (42.22)	
Combined approach	75 (36.23)	49 (30.25)	26 (57.78)	
Lateral malleolus osteotomy [N(%)]				0.844
No	201 (97.10)	158 (97.53)	43 (95.56)	
Yes	6 (2.90)	4 (2.47)	2 (4.44)	
Medial malleolus osteotomy [N(%)]				0.072
No	187 (90.34)	150 (92.59)	37 (82.22)	
Yes	20 (9.66)	12 (7.41)	8 (17.78)	
Intraoperative blood loss (mL)	52.56 ± 47.16	45.19 ± 34.60	79.11 ± 71.50	<0.001^*^
Operating duration (min)	90.22 ± 36.02	85.00 ± 34.85	109.00 ± 34.17	<0.001^*^
Postoperative reduction [N(%)]				<0.001^*^
Nearly anatomical	180 (86.96)	155 (95.68)	25 (55.56)	
Poor	27 (13.04)	7 (4.32)	20 (44.44)	
Follow up (month)	16.53 ± 5.32	16.35 ± 5.47	17.18 ± 4.74	0.231
Fixation removal [N(%)]				0.113
No	182 (87.92)	146 (90.12)	36 (80.00)	
Yes	25 (12.08)	16 (9.88)	9 (20.00)	

*P < 0.05, with statistical significance.

### 3.2 Univariate and multivariable logistic regression

Univariate logistic regression analysis identified several variables significantly associated with the development of AVN following talar fractures, including operating duration, intraoperative blood loss, BMI, fracture classification, surgical approach, medial malleolus osteotomy, fixation removal, concomitant ipsilateral foot and ankle fractures, smoke, surgical strategy, quality of fracture reduction, and fracture type. Multivariable logistic regression further confirmed six independent risk factors: BMI, fracture classification, concomitant ipsilateral foot and ankle fractures, smoking, quality of fracture reduction, and fracture type (Table 3).

**TABLE 3:** Univariate logistic regression analysis and multivariate logistic regression analysis.

Variable	Univariate analysis				Multivariate analysis			
	OR	95%CI (Lower)	95%CI (Upper)	P value	OR	95%CI (Lower)	95%CI (Upper)	*P* value
Age	1.000	0.995	1.004	0.847				
Gender	0.880	0.761	1.017	0.146				
ASA class	0.974	0.829	1.145	0.785				
BMI	1.038	1.021	1.054	<0.001^*^	1.018	1.006	1.031	0.017^*^
Hypertension	1.003	0.858	1.171	0.979				
Diabetes	0.801	0.567	1.133	0.291				
Heart disease	1.037	0.809	1.331	0.807				
Smoke	1.495	1.335	1.674	<0.001^*^	1.175	1.063	1.298	0.009^*^
Alcohol	1.112	0.952	1.298	0.260				
Injury mechanism	1.016	0.906	1.140	0.820				
Fracture side	0.986	0.880	1.105	0.841				
Fracture classification	1.082	1.060	1.105	<0.001^*^	1.029	1.009	1.050	0.018^*^
Fracture type	2.001	1.680	2.382	<0.001^*^	1.395	1.169	1.667	0.002^*^
Concomitant ipsilateral foot and ankle fractures	1.400	1.261	1.554	<0.001^*^	1.120	1.022	1.229	0.042^*^
Time to surgery	0.996	0.982	1.009	0.608				
Surgical strategy	1.704	1.298	2.239	0.001^*^	1.164	0.922	1.470	0.282
Fixation method	1.033	0.986	1.082	0.252				
Surgical approach	1.182	1.052	1.327	0.019^*^	1.053	0.954	1.161	0.388
Lateral malleolus osteotomy	0.984	0.720	1.344	0.933				
Medial malleolus osteotomy	1.311	1.078	1.594	0.023^*^	1.127	0.969	1.313	0.195
Intraoperative blood loss	1.003	1.001	1.004	<0.001^*^	0.999	0.998	1.000	0.105
Operating duration	1.003	1.001	1.004	0.002^*^	1.001	0.999	1.002	0.510
Postoperative reduction	1.830	1.600	2.094	<0.001^*^	1.419	1.239	1.624	<0.001^*^
Follow up	1.002	0.991	1.012	0.818				
Fixation removal	1.336	1.124	1.587	0.006^*^	0.980	0.857	1.120	0.801

*P < 0.05, with statistical significance.

### 3.3 Model building and performance evaluation

**FIGURE 1:**
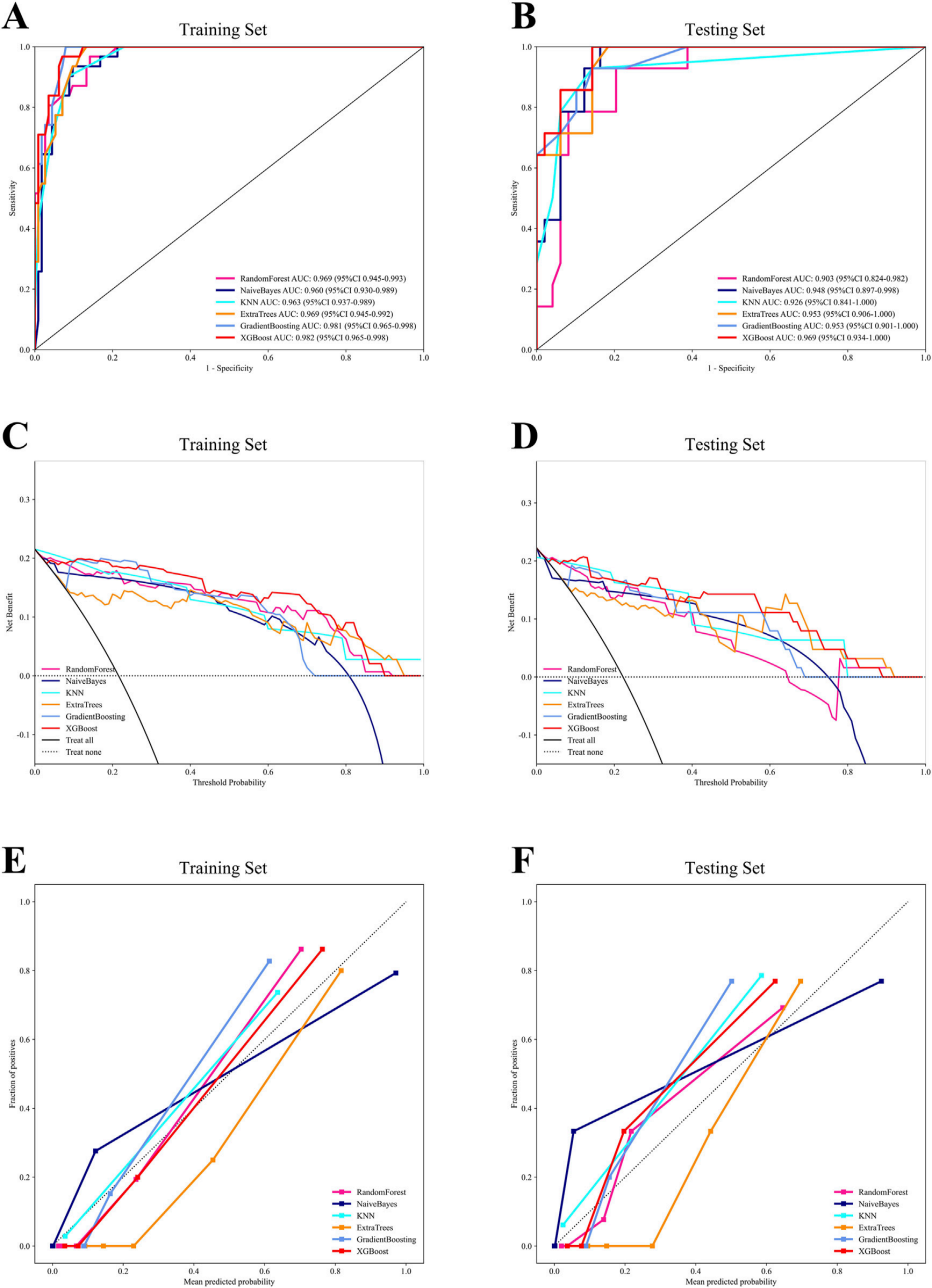
The comprehensive analysis of six machine learning models. **(A)** The ROC curve of the training set. **(B)** The ROC curve of the testing set. **(C)** The DCA curve of the training set. **(D)** The DCA curve of the testing set. **(E)** The calibration curve of the training set. **(F)** The calibration curve of the testing set.

**FIGURE 2:**
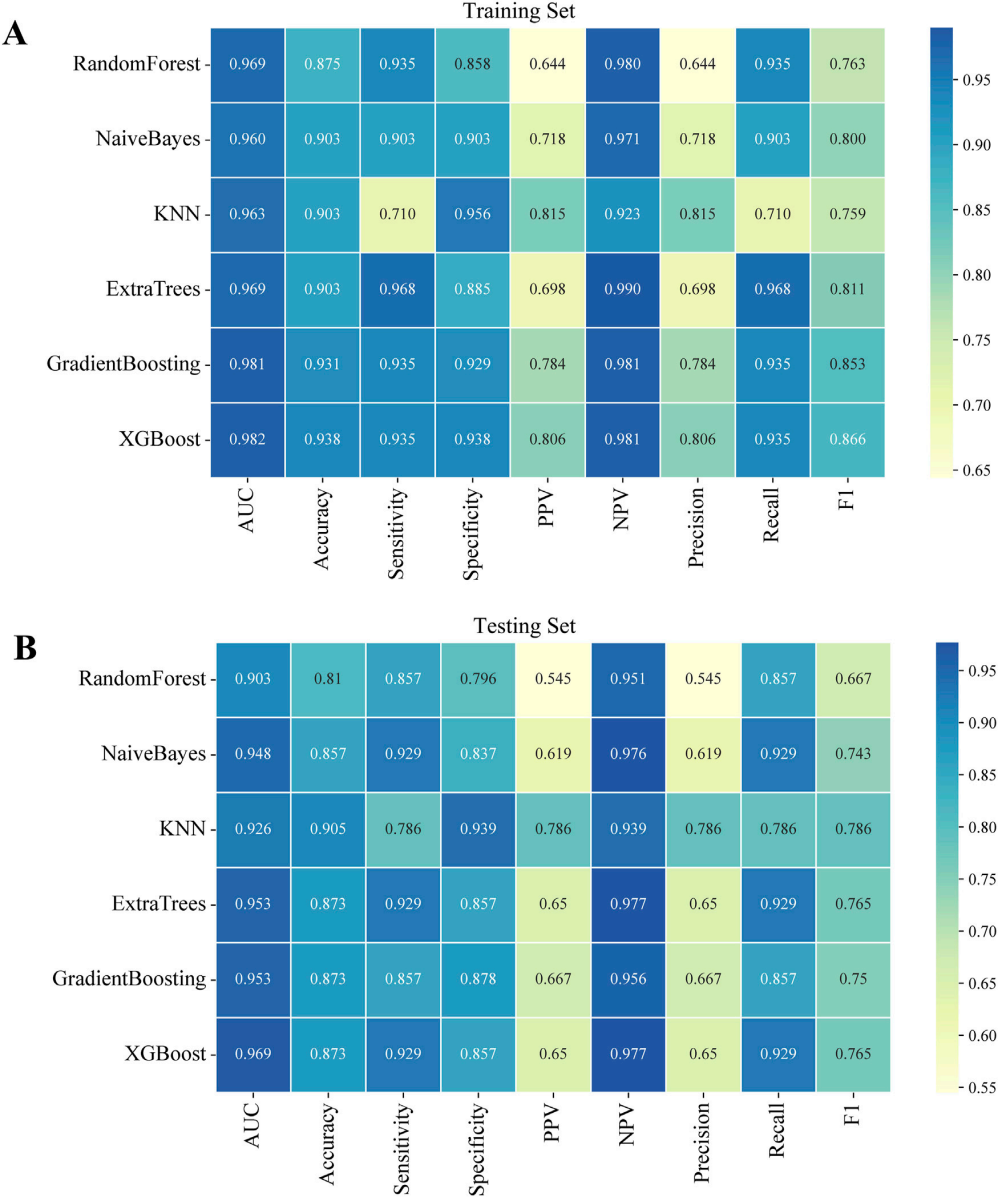
Performance indicators of six machine learning models in both the training and testing sets. **(A)** The training set. **(B)** The testing set.

Using the six independent risk factors identified by multivariable logistic regression, we constructed six machine learning models in the training set. Predictive performance was evaluated using five-fold cross-validation and assessed with metrics including AUC, calibration curves, and DCA. The results of AUC demonstrated that all models exhibited outstanding predictive performance, with XGBoost achieving the highest diagnostic accuracy in both the training and testing sets (Figures 1A,B). Additionally, XGBoost showed the best performance in terms of calibration curves and DCA curves, indicating superior calibration and clinical applicability (Figures 1C–F). To comprehensively evaluate model performance, we calculated additional metrics, including accuracy, sensitivity, specificity, PPV, NPV, precision, recall, and F1 score for all six machine learning models in both the training and testing sets (Figures 2A,B). Based on the combined evaluation of all metrics, XGBoost was the most accurate and reliable for predicting AVN following talar fractures.

**FIGURE 3:**
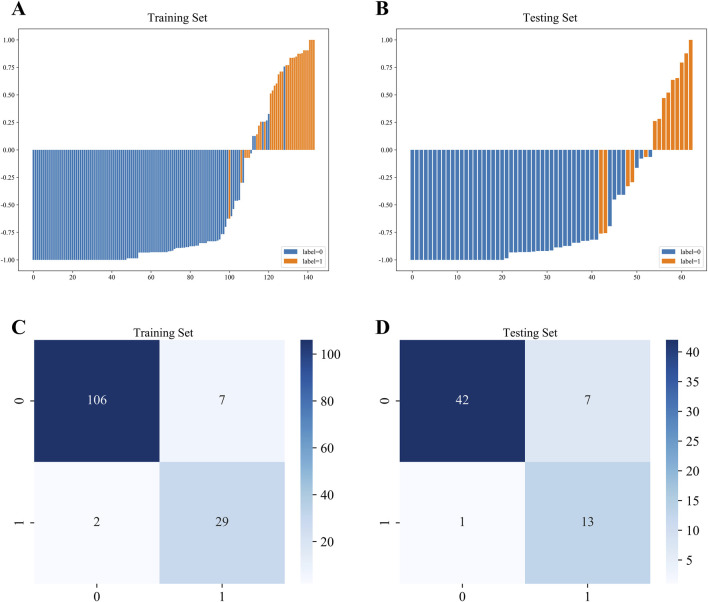
Waterfall chart and confusion matrix of XGBoost model. **(A)** Waterfall chart of the training set. **(B)** Waterfall chart of the testing set. **(C)** Confusion matrix of the training set. **(D)** Confusion matrix of the testing set.

Waterfall charts demonstrated that XGBoost model exhibited strong predictive performance in both the training and testing sets, as shown in Figures 3A,B. Additionally, confusion matrices were constructed to evaluate the model’s performance and transparency in predicting AVN (Figures 3C,D). The results revealed that XGBoost model achieved excellent predictive accuracy, with high sensitivity and specificity in both datasets.

### 3.4 Model explanation

**FIGURE 4:**
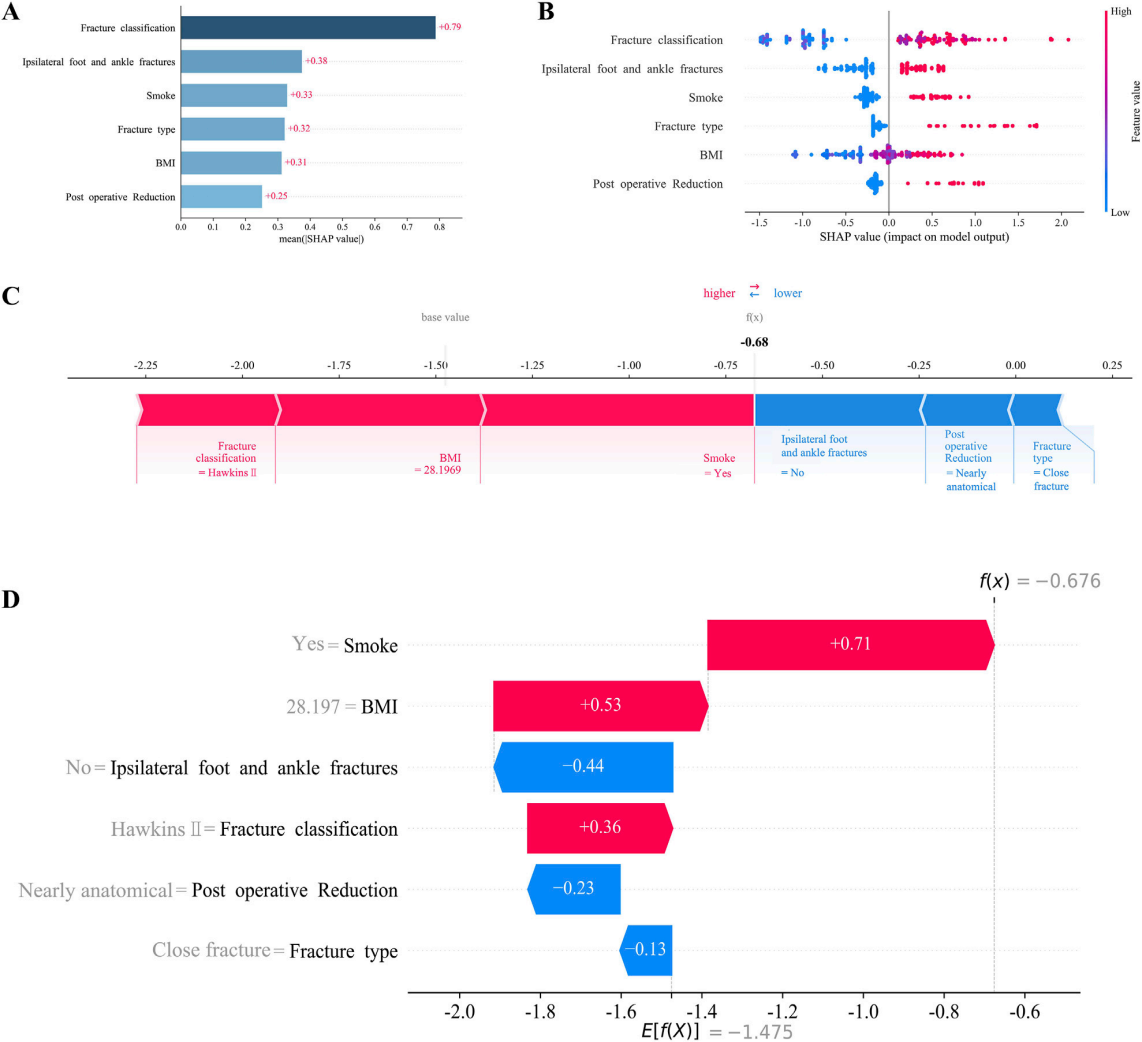
Interpretation of XGBoost model using the SHAP. **(A)** Importance ranking of features displayed by the SHAP. **(B)** Characterization attributes in the SHAP. **(C)** Examples of explicable outcomes of a patient suffering from AVN following talar fractures. **(D)** The SHAP values of a patient suffering from AVN following talar fractures.

To enhance clinical interpretability, we utilized the SHAP method to explain the final XGBoost model. This approach provided two types of explanations: global explanations of the model at the feature level and local explanations at the individual level. Global explanations, illustrated in the SHAP summary plot, ranked the features based on their contribution to the model using the SHAP mean values. Smoking, BMI, and concomitant ipsilateral foot and ankle fractures were identified as the three most important predictors of AVN (Figure 4A). In addition, the SHAP dependence plot illustrated the influence of individual features on model predictions, with red representing high risk values and blue representing low risk values (Figure 4B). In SHAP analysis, positive SHAP values for features such as smoking and BMI indicate an elevated risk of AVN, whereas negative values suggest a protective effect. For instance, higher BMI values are associated with increased AVN risk, attributable to greater mechanical stress and metabolic disturbances. Conversely, certain fracture classifications with negative SHAP values correlate with reduced AVN risk, likely reflecting lesser fracture displacement and diminished vascular compromise.

For local explanations, we analyzed specific patients to understand how their individual characteristics contributed to the prediction of AVN. Figures 4C,D illustrated the SHAP force plot for a patient who developed AVN. Red features indicated a facilitating effect on the occurrence of AVN. On the contrary, blue features represented an inhibitory effect, and the length of the arrow represents the magnitude of the feature’s contribution.

## 4 Discussion

This study successfully developed and validated a prediction model for AVN following talar fractures by applying machine learning. We identified BMI, fracture classification, concomitant ipsilateral foot and ankle fractures, smoke, quality of fracture reduction, and fracture type as key risk factors for AVN. The XGBoost model demonstrated robust discriminatory and calibration capabilities, providing valuable clinical guidance and highlighting the potential of machine learning for predicting orthopedic postoperative complications.

Our findings underscore the critical role of smoking as the most influential predictor in the model, attributable to its detrimental effects on vascular endothelial function and local blood supply (Patel et al., 2013). Smoking induces vasospasm, thrombosis, and microcirculatory disturbances, reducing blood supply and increasing the risk of AVN following talar fractures (Kondo et al., 2019). The result was consistent with previous studies and emphasized the critical importance of preoperative smoking cessation, especially for talar fracture patients.

Patients with high BMI often exhibit obesity-related metabolic dysregulation and chronic inflammation, potentially disrupting the microenvironment necessary for fracture healing. Fang et al. reported that the incidence of hyperlipidemia is significantly higher in high BMI patients, and hyperlipidaemia increases the risk of AVN by forming fat plugs that hinder neovascularisation (Pei et al., 2020). In addition, elevated BMI may increase local mechanical stress on the talus, potentially raising the risk of fracture displacement (Collins et al., 2018). Our finding highlighted the need for comprehensive preoperative assessment and targeted weight management strategies in high BMI patients to optimize outcomes and minimize the risk of AVN.

Concomitant ipsilateral foot and ankle fractures, typically indicative of higher-energy trauma, further compromise the talus’s blood supply and surrounding soft tissues (Srinath et al., 2024). In addition, ipsilateral foot and ankle fractures may limit postoperative rehabilitation activities, indirectly impairing blood circulation. Zhang et al. reported that inflammatory markers and osteoclast activity were elevated in multiple fractures compared with single fractures (Zhang et al., 2021). Furthermore, Zheng et al. suggested that the chronic inflammatory microenvironment regulated by bone immune abnormalities may contribute significantly to AVN pathogenesis (Zheng et al., 2022). Our findings emphasized the importance of recognizing and managing comorbid injuries in patients with talar fractures, as they necessitate tailored surgical and rehabilitation protocols to mitigate the risk of AVN.

Fracture type and classification reflect injury severity and anatomical disruption (Jordan et al., 2017). Our results demonstrate a higher incidence of AVN in patients with talar neck combined with body fracture, potentially enhancing the prognostic utility of the Hawkins and Sneppen classification system (Vallier et al., 2014; Mechas et al., 2023). Additionally, the quality of fracture reduction emerged as a significant predictor of AVN, as anatomical reduction maximizes the restoration of blood circulation around the talus. We defined poor reduction as >2 mm displacement or >5° neck angulation, consistent with the research of Biz (Biz et al., 2019). This underscores the critical importance of meticulous surgical technique and appropriate fixation to achieve and maintain optimal reduction, thereby reducing the risk of AVN.

The application of machine learning in this study demonstrates its tremendous capabilities in orthopaedics. By leveraging multidimensional clinical data, machine learning models can automatically identify complex data patterns and provide personalized predictions, offering advantages over traditional statistical methods in handling nonlinear relationships and high-dimensional data. Despite promising results, this study has certain limitations. As a retrospective study, the reliance on medical records may introduce limitations in data quality and reduce the credibility of the evidence. Additionally, the performance of machine learning models is contingent on the diversity and representativeness of the training data, and our single-center study design may limit model generalizability. Performance may vary across institutions due to surgical technique heterogeneity or demographic differences. Therefore, future multicenter prospective studies with larger samples are warranted to enhance generalizability and clinical applicability. Furthermore, self-reported factors like smoking and alcohol use are susceptible to reporting bias. BMI and smoking may proxy unmeasured confounders such as hyperlipidemia or sedentary behavior. Although SHAP quantifies feature contributions, residual confounding could bias interpretations. Hence, incorporating serological markers such as lipid profiles and objective lifestyle measures with rigorous follow-up protocols would further validate the reliability of the model.

## 5 Conclusion

In conclusion, our study developed a novel predictive framework for AVN following talar fractures, leveraging machine learning to identify key risk factors and assess their contributions to the development of this complication. The findings advance our understanding of the pathophysiology of AVN and offer practical insights for clinicians to optimize surgical planning and postoperative management. However, due to the lack of external validation of the present study, future multicenter validation and refinement are warranted to ensure broader clinical applicability and effectiveness.

## Data Availability

The raw data supporting the conclusions of this article will be made available by the authors, without undue reservation.

## References

[B1] Alley M. C. Vallier H. A. Tornetta P. 3rd (2024). Identifying risk factors for osteonecrosis after talar fracture. J. Orthop. Trauma 38 (1), 25–30. 10.1097/bot.0000000000002706 37735752

[B2] Biz C. Golin N. De Cicco M. Maschio N. Fantoni I. Frizziero A. (2019). Long-term radiographic and clinical-functional outcomes of isolated, displaced, closed talar neck and body fractures treated by ORIF: the timing of surgical management. BMC Musculoskelet. Disord. 20 (1), 363. 10.1186/s12891-019-2738-2 31391024 PMC6686493

[B3] Chen H. Liu W. Deng L. Song W. (2014). The prognostic value of the hawkins sign and diagnostic value of MRI after talar neck fractures. Foot Ankle Int. 35 (12), 1255–1261. 10.1177/1071100714547219 25116131

[B4] Choi J. Y. Kim H. S. Ngissah R. Suh J. S. (2023). Operative outcomes of a high-grade talar neck fracture - lessons from 20 years' clinical experience in a single, tertiary hospital. Foot Ankle Surg. 29 (2), 118–127. 10.1016/j.fas.2022.12.002 36526523

[B5] Churpek M. M. Carey K. A. Edelson D. P. Singh T. Astor B. C. Gilbert E. R. (2020). Internal and external validation of a machine learning risk score for acute kidney injury. JAMA Netw. Open 3 (8), e2012892. 10.1001/jamanetworkopen.2020.12892 32780123 PMC7420241

[B6] Collins A. T. Kulvaranon M. L. Cutcliffe H. C. Utturkar G. M. Smith W. A. R. Spritzer C. E. (2018). Obesity alters the *in vivo* mechanical response and biochemical properties of cartilage as measured by MRI. Arthritis Res. Ther. 20 (1), 232. 10.1186/s13075-018-1727-4 30333058 PMC6235204

[B7] Dodd A. Lefaivre K. A. (2015). Outcomes of talar neck fractures: a systematic review and meta-analysis. J. Orthop. Trauma 29 (5), 210–215. 10.1097/bot.0000000000000297 25635362

[B8] Du S. Wu Y. Tao J. Shu L. Yan T. Xiao B. (2025). Development and validation of machine learning models for outcome prediction in patients with poor-grade aneurysmal subarachnoid hemorrhage following endovascular treatment. Ther. Clin. Risk Manag. 21, 293–307. 10.2147/tcrm.S504745 40071129 PMC11895686

[B9] Fanizzi A. Arezzo F. Cormio G. Comes M. C. Cazzato G. Boldrini L. (2024). An explainable machine learning model to solid adnexal masses diagnosis based on clinical data and qualitative ultrasound indicators. Cancer Med. 13 (12), e7425. 10.1002/cam4.7425 38923847 PMC11196372

[B10] Harris A. H. Kuo A. C. Bowe T. Gupta S. Nordin D. Giori N. J. (2018). Prediction models for 30-Day mortality and complications after total knee and hip arthroplasties for veteran health administration patients with osteoarthritis. J. Arthroplasty 33 (5), 1539–1545. 10.1016/j.arth.2017.12.003 29398261 PMC6508537

[B11] Haug C. J. Drazen J. M. (2023). Artificial intelligence and machine learning in clinical medicine, 2023. N. Engl. J. Med. 388 (13), 1201–1208. 10.1056/NEJMra2302038 36988595

[B12] Hu J. Xu J. Li M. Jiang Z. Mao J. Feng L. (2024). Identification and validation of an explainable prediction model of acute kidney injury with prognostic implications in critically ill children: a prospective multicenter cohort study. EClinicalMedicine 68, 102409. 10.1016/j.eclinm.2023.102409 38273888 PMC10809096

[B13] Jordan R. K. Bafna K. R. Liu J. Ebraheim N. A. (2017). Complications of talar neck fractures by hawkins classification: a systematic review. J. Foot Ankle Surg. 56 (4), 817–821. 10.1053/j.jfas.2017.04.013 28633784

[B14] Karnuta J. M. Haeberle H. S. Luu B. C. Roth A. L. Molloy R. M. Nystrom L. M. (2021). Artificial intelligence to identify arthroplasty implants from radiographs of the hip. J. Arthroplasty 36 (7s), S290–S294.e1. 10.1016/j.arth.2020.11.015 33281020

[B15] Kondo T. Nakano Y. Adachi S. Murohara T. (2019). Effects of tobacco smoking on cardiovascular disease. Circ. J. 83 (10), 1980–1985. 10.1253/circj.CJ-19-0323 31462607

[B16] Kubisa M. J. Kubisa M. G. Pałka K. Sobczyk J. Bubieńczyk F. Łęgosz P. (2024). Avascular necrosis of the talus: diagnosis, treatment, and modern reconstructive options. Med. Kaunas. 60 (10), 1692. 10.3390/medicina60101692 PMC11509827 39459479

[B17] Li L. Yang X. Guo W. Wu W. Guo M. Li H. (2025). Predicting the risk of postoperative gastrointestinal bleeding in patients with type A aortic dissection based on an interpretable machine learning model. Front. Med. (Lausanne) 12, 1554579. 10.3389/fmed.2025.1554579 40458646 PMC12127380

[B18] Mechas C. A. Aneja A. Nazal M. R. Pectol R. W. Sneed C. R. Foster J. A. (2023). Association of talar neck fractures with body extension and risk of avascular necrosis. Foot Ankle Int. 44 (5), 392–400. 10.1177/10711007231160751 36999214

[B19] Patel R. A. Wilson R. F. Patel P. A. Palmer R. M. (2013). The effect of smoking on bone healing: a systematic review. Bone Jt. Res. 2 (6), 102–111. 10.1302/2046-3758.26.2000142 PMC3686151 23836474

[B20] Pei F. Zhao R. Li F. Chen X. Guo K. Zhu L. (2020). Osteonecrosis of femoral head in young patients with femoral neck fracture: a retrospective study of 250 patients followed for average of 7.5 years. J. Orthop. Surg. Res. 15 (1), 238. 10.1186/s13018-020-01724-4 32600432 PMC7322831

[B21] Quan K. R. Lin W. R. Hong J. B. Lin Y. H. Chen K. Q. Chen J. H. (2024). A machine learning approach for predicting radiation-induced hypothyroidism in patients with nasopharyngeal carcinoma undergoing tomotherapy. Sci. Rep. 14 (1), 8436. 10.1038/s41598-024-59249-3 38600141 PMC11006930

[B22] Saravi B. Lang G. Ruff R. Schmal H. Südkamp N. Ülkümen S. (2021). Conservative and surgical treatment of talar fractures: a systematic review and meta-analysis on clinical outcomes and complications. Int. J. Environ. Res. Public Health 18 (16), 8274. 10.3390/ijerph18168274 34444022 PMC8393919

[B23] Srinath A. Southall W. G. S. Nazal M. R. Mechas C. A. Foster J. A. Griffin J. T. (2024). Talar neck fractures with associated ipsilateral foot and ankle fractures have a higher risk of avascular necrosis. J. Orthop. Trauma 38 (6), 220–224. 10.1097/bot.0000000000002798 38457751

[B24] Vallier H. A. Reichard S. G. Boyd A. J. Moore T. A. (2014). A new look at the hawkins classification for talar neck fractures: which features of injury and treatment are predictive of osteonecrosis? J. Bone Jt. Surg. Am. 96 (3), 192–197. 10.2106/jbjs.L.01680 24500580

[B25] Wang Z. Sun Z. Yu L. Wang Z. Li L. Lu X. (2023). Machine learning-based prediction of composite risk of cardiovascular events in patients with stable angina pectoris combined with coronary heart disease: development and validation of a clinical prediction model for Chinese patients. Front. Pharmacol. 14, 1334439. 10.3389/fphar.2023.1334439 38269285 PMC10806135

[B26] Zhang C. Zhu J. Jia J. Guan Z. Sun T. Zhang W. (2021). Effect of single *versus* multiple fractures on systemic bone loss in mice. J. Bone Min. Res. 36 (3), 567–578. 10.1002/jbmr.4211 33181861

[B27] Zheng J. Yao Z. Xue L. Wang D. Tan Z. (2022). The role of immune cells in modulating chronic inflammation and osteonecrosis. Front. Immunol. 13, 1064245. 10.3389/fimmu.2022.1064245 36582244 PMC9792770

