# Supplementary material for: Predicting the risk of postoperative avascular necrosis in patients with talar fractures based on an interpretable machine learning model
Source: Front Bioeng Biotechnol. 2025 Jul 31;13:1644261. doi: 10.3389/fbioe.2025.1644261 (PMC12351182; doi:10.3389/fbioe.2025.1644261)
Supplement: Supplementary file 1 [file Table1.docx]

| **Supplementary Table 1.** Final hyperparameters setting of the six models. |
| --- |
| **new_models[’RandomForest’]** = RandomForestClassifier(n_estimators=10, max_depth=3, min_samples_split=2, random_state=0); |
| **new_models[’NaiveBayes’]** = GaussianNaiveBayes(); |
| **new_models[’GradientBoosting’]** = GradientBoostingClassifier(n_estimators=10, random_state=0, max_depth=3); |
| **new_models[’K-NearestNeighbors’]** = K-NearestNeighborsClassifier(algorithm=’kd_tree’); |
| **new_modelsl[’ExtraTrees’]** = ExtraTreesClassifier(class_weight =’balanced’, n_estimators=10,max_depth=3, min_samples_split=2, random_state=0); |
| **new_models[’XGBoost’]** = XGBoostClassifier (scale_pos_weight=1, n_estimators=10, objective=’binary:logistic’, max_depth=3, use_label_encoder=False, eval_metric=’error’). |
